# The Death of Baron Liebig

**Published:** 1873-07

**Authors:** Charles A. Joy


*The Death of Baron Liebig.* By PROFESSOR CHARLES A. JOY.

The death of Baron Liebig will create a profound sensation throughout the civilized world. No scientific man was better known, and none had rendered a greater service to the popularization of knowledge than he. Wherever books are read and journals are printed, his name has become familiar as a household word, and his opinion has always been regarded as worthy of the highest authority. The part played by this distinguished chemist is worthy of something more than a passing notice, and I propose to address myself to the task of giving some account of what he has done.

Justus Liebig was born in Darmstadt, May 13, 1803. His parents were poor, and, if it had not been for Government aid, he could never have sustained himself at school, but would have been compelled to learn a trade. The boy early developed a taste for natural history, and his father encouraged this indication by permitting him to study under the direction of a pharmacist in a neighboring town, but a desire to obtain a more thorough education, prompted young Liebig to apply to the Minister of Instruction for assistance from the public fund. I have heard him relate his experience with this high official. When he applied at the palace, it was only after repeated efforts that he was permitted an audience, and, at the first interview, the minister declined to grant the request; nothing daunted, he kept on repeating his calls until the officer, in self-defense, was compelled to put the boy on the stipend. In this way Liebig gained admittance to the Gymnasium, and was able to go through the preparatory course for admission to the University. While at the Gymnasium, he and another boy fell into considerable discredit with the director for presuming to develop tastes for something besides the classics. The director was in the habit of asking the boys what they intended to be if they lived to grow up, and when he came to Liebig, he invariably received the answer: "I intend to be a chemist;" whereupon the pedagogue would say, "You stupid boy, there is no such pursuit as chemist;" to the other lad, who desired to be a musician, he could hardly give a similar answer, although he assigned to both of them places at the foot of the roll. Liebig told me that many years afterwards he came across his companion in disgrace, and found him to be the most popular musical director in the city of Vienna.

Having completed the course at the Gymnasium, Liebig studied at Bonn and Erlangen, and subsequently received the degree of Doctor of Philosophy and Doctor of Medicine. In the early part of 1823, when only twenty years of age, he went to Paris, hoping to obtain access to the laboratory of Gay-Lussac (born 1778, died 1850); it was a hazardous thing for a poor student to undertake, but he felt that work was in him if he could secure an opportunity to bring it out. The early stages of his career in Paris were the most trying of his life. Without money, without patronage, with nothing but youth and a resolute will to sustain him, his case appeared to be forlorn in the extreme, and he was wont to say that his whole career might have been cut short, and his name never have become known, if it had not been for the fortunate acquaintance of Alexander Von Humboldt, formed at a meeting of the French Institute. Gay-Lussac was much interested in the subject of cyanogen, and young Liebig had prepared compounds analogous to the cyanides, in which he thought he had discovered a new acid. One of the members of the Institute promised to read his paper on the subject and exhibit the specimens, but, like the Minister of Instruction at Darmstadt, it was only after repeated warnings and entreaties that he consented to keep his engagement. The subject was finally presented, and attracted considerable notice. After the adjournment, a gentleman whom Liebig did not know, came up to the table to speak to him and to inquire into his history, and, being pleased with his intelligence and modest deportment, invited him to dinner.

Young Liebig was too much embarrassed to ask the name of his host, and as he left the house inquired of the doorkeeper who that gentleman was. The doorkeeper replied that a good many persons had passed out, and he could not say to which particular one he referred; moreover, as Alexander Von Humboldt had just returned to Paris from a long journey, very few persons were aware of his presence in the city. Liebig could not keep his appointment with Humboldt, as he did not know where to go, but as soon as he learned the name of his benefactor he at once called on him, and from this acquaintance with Humboldt he dates the commencement of his prosperity. Upon Humboldt's recommendation, Gay-Lussac received Liebig as a pupil in his laboratory, and the two remained ever afterwards firm friends. The fame thus early acquired by Liebig soon reached his native land, and he was invited in 1824, at the early age of twenty-one, to accept the chair of chemistry at the University of Giessen. This university was destined to become the principal field of his usefulness and renown. It was here that he established his celebrated school of chemistry, and it was in the laboratory, built under his direction, that he made the researches which have so greatly enriched our knowledge and been of such vast importance to the well-being of mankind. At this early period there was no school for chemistry in the world. There were private laboratories where a few favored students could obtain instruction, the most celebrated of which was in Sweden, under the direction of Berzelius, but such an institution as a school for chemistry was unknown until founded by Liebig. If Liebig had conferred no other benefit upon the world than this one act of establishing a systematic course of instruction in chemistry, it would have been sufficient to establish his claim to a prominent place on the list of benefactors of the race. The school was a means to an end, and enabled him to carry out the investigations and researches which have proved of so much importance to the progress of the science. The laboratory of Giessen became a hive of busy workers. Hoffmann, Strecker, Will, Fesenius, Whitney, Gibbs, Horsford, and a host of others, here laid the foundations of their knowledge and subsequent fame. The Giessen school served as a model for similar institutions all over the world, and the way having been once pointed out, it was rapidly followed and considerably improved as the years rolled by. Plans of the laboratory were engraved to supply the constant demand for them, and architects were sent from many countries to study the new arrangements preparatory to their construction, at distant universities and schools. Although numerous modifications have been suggested by experience, yet, upon the whole, the original plan of Liebig has been retained, in a majority of laboratories, with such changes as modifications in the construction of furnaces, the introduction of gas and improvements in apparatus, may have required. There is no question that while laboratory practice was of great value to students, the work accomplished by some of the more advanced scholars was of equal service to Liebig in enabling him to institute numerous researches which it would have been physically impossible for him to accomplish alone. It was sometimes necessary to make a large number of analyses in order to ascertain the accuracy of methods or to prove the correctness of theories, and in the early stages of chemical research, much more of this work was required than at the present time. In the course of time a rich store of observations was accumulated at the Giessen laboratory, which became available to chemists elsewhere, and this supply of material has since been largely added to by other laboratories in various parts of the world.

Liebig's industry in the preparation of original papers and in the publication of books was remarkable. The number of independent articles published in various journals does not vary far from 350, and his books, from his "Introduction to Organic Analysis" down to the last edition of his great work on "Agricultural Chemistry," number about thirty volumes. In addition to this, he did a great deal of editorial work upon Geiger's Handbook, the yearly reports on the progress of chemistry, the annals of chemistry and pharmacy, and the scientific journals of the day.

The great aim of Liebig was to popularize science. To this end, he wrote his famous "Letters on Chemistry," which first appeared as an extra sheet in a weekly newspaper, and were afterwards collected in book form, and translated into every European language. No letters have been more generally read or have excited greater popular enthusiasm than these. They are perpetually quoted and criticized, and thus their subject matter has been presented in a great variety of shapes, until the views originally held by Liebig have been unconsciously everywhere adopted, although he himself greatly modified some of them, as subsequent research shed new light upon the subject. It has been said that Liebig was very hasty in drawing conclusions from imperfect premises. This may have been in a measure true; and when we consider how numerous these theories were, it is surprising that more of them were not overthrown. If Liebig was hasty in expressing an opinion, he was equally ready to retract when convinced that it was erroneous. I once had a conversation with him on this point, and he spoke of the fear some scientific men had of being caught in a mistake. He considered errors inevitable and inseparable from the accomplishment of any scientific work, and it was on this occasion that he made the oft-quoted remark, "show me the man who makes no mistakes, and I will prove to you that he does nothing." It is easy enough for a scientific man who publishes nothing, but only criticizes others, to escape the charge of committing errors, and for such persons Liebig entertained a wholesome contempt. What he desired, above all things, to know, was the truth, and when he thought he had discovered it, he was anxious to give the benefit of it to the world. It would occupy too much space to undertake to give a history of the work accomplished by this great man. The chief points of it can, however, be compressed into a small compass. He pointed out the way to conduct the analysis of organic bodies, and to establish for them rational formulas. In conjunction with Woehler he accomplished the synthesis of organic bodies, and thus did what Berzelius pronounced to be impossible. He introduced chemistry into the study of physiology, and thus created a revolution in that branch of science, and in the department of agriculture did more to place that pursuit upon a scientific basis than any man who had preceded him.

Pharmacy, physiology, agricultural and synthetical chemistry, owe a tremendous debt of gratitude to the illustrious Liebig. After devoting nearly thirty years unremittingly to the Giessen school, Liebig received a call from the King of Bavaria to go to Munich. In 1852, on the occasion of a visit I made to him, in company with Professor Henry Rose, I found him engaged in packing up his papers, preparatory to removal to Munich. His favorite pupil, Professor Hoffmann, now in Berlin, was assisting him in arranging his letters, and on the occasion of our visit Liebig read to us a number of letters he had received from Berzelius, which showed the sincere respect the great Swedish chemist entertained for the man who had not been his pupil, but whom he looked upon as a most worthy colleague. Liebig subsequently accompanied us to his laboratory, where he exhibited to Rose a number of delicate tests he had just discovered. Liebig was very loth to leave Giessen. The inhabitants had shown their appreciation of his worth by presenting him a valuable house and garden, and it was here that his children were born and his fame as a chemist had been established. The necessity for such rest as could only be attained by a change of residence was, however, fully impressed upon him, and he decided to accept the invitation of the King of Bavaria. After going to Munich, Liebig only received such pupils as could aid him in his researches. The work that chiefly occupied him, was a careful revision of his book on Agricultural Chemistry, a controversy with Pasteur on the subject of fermentation, and the introduction of extract of beef, as a cheap food for the poor. A desire to benefit his fellowmen characterized the labors of Liebig during the last years of his life. The study of a method of silvering glass was instituted out of a wish to rescue from disease the poor victims of mercury poisoning, who were engaged in the manufacture of quicksilver mirrors. The extract of meat he hoped would secure a cheap and wholesome food for the poor, and furnish an invaluable remedy for invalids and convalescents. Latterly his mind was full of similar schemes of beneficence, and his last publications all look to the dissemination of correct scientific information on the common affairs of life. It ought not to be forgotten, in this connection, that Liebig was the first person in Europe to prepare chloroform, and it was he who discovered the hydrate of chloral—the two great anæsthetic and hypnotic agents now so largely employed to alleviate the sufferings of mankind.

In his personal relations I have always found Liebig to be full of urbanity and hospitality. I am aware that many Germans complained of his imperious and haughty manner, and he was often assailed by disappointed chemists on this score, but to any one really in earnest, he always appeared to me ever ready to lend a helping hand. He was a man who kept up with the literature of the day, and could converse fluently and well on any of the topics likely to be presented in the high circles in which he moved. Every summer he was in the habit of going to some quiet place among the mountains for relaxation, and on these occasions always contrived to make up a whist party of the most distinguished professors in Germany. I once spent a week with one of these parties when it was composed of Liebig, Woehler, Clausius, Schoenbein, and Hans Christian Andersen, and it was pleasant to see men of such renown in their moments of rest and recreation. The liveliest of them all was Schoenbein, but Liebig was equal to any of them in repartee and anecdote. It was on this occasion that Liebig related to me many of the incidents of his life which are recorded above. The scientific men who were the contemporaries and fellow workers with Liebig, have nearly all passed away. Henry Rose, Mitscherlich, Erdmann, Magnus, Gmelin, Haidinger, Schoenbein, Faraday, Oersted, Pelouze, have ceased their labors, and there remain Woehler, Gustavus Rose, Poggendorff, Mulder, Dumas—not lingering superfluously on the stage, but still actively engaged in the promotion of scientific knowledge.

A new generation is springing up, to whom Liebig has left a legacy of inestimable value in the example he has set of obstacles overcome, of work accomplished, and of benefits rendered to his fellowmen. At the good old age of nearly three score years and ten he has been gathered to his fathers, honored and loved by all who knew him.—*Journal of Applied Chemistry.*

